# Slope reducing high tibial osteotomy and revision anterior cruciate ligament reconstruction leads to satisfying clinical results and a low failure rate

**DOI:** 10.1002/jeo2.70260

**Published:** 2025-05-19

**Authors:** Lorenz Fritsch, Konstantin Dworschak, Maximilian Hinz, Philipp W. Winkler, Bastian Scheiderer, Sebastian Siebenlist, Romed Vieider, Lukas Willinger, Stefan Hinterwimmer, Julian Mehl

**Affiliations:** ^1^ Department of Sports Orthopaedics Technical University of Munich Munich Germany; ^2^ The Steadman Philippon Research Institute Vail Colorado USA; ^3^ Department for Orthopaedics and Traumatology Kepler University Hospital GmbH, Johannes Kepler University Linz Linz Austria

**Keywords:** anterior closing wedge osteotomy, anterior cruciate ligament reconstruction, clinical outcome, posterior tibial slope, revision, slope‐reducing osteotomy

## Abstract

**Purpose:**

This study aimed to assess clinical and radiological outcomes after two‐staged slope‐reducing high tibial osteotomy (HTO) and revision anterior cruciate ligament reconstruction (ACLR) for recurrent ACL insufficiency with an increased posterior tibial Slope (PTS) > 12°.

**Methods:**

Patients operated in two centres between 01/2015 and 01/2022 were included after a minimum follow‐up of 24 months after revision ACLR. The postoperative pain and the following scores were obtained: IKDC, KOOS, Lysholm, TAS. The Slope was measured using the Dejour technique postoperatively using lateral X‐rays being compared to a preoperative X‐ray. Also, a clinical examination including range of motion, anterior + posterior translation and pivot‐shift were performed. The Rolimeter was used for anterior tibial translation (ATT).

**Results:**

Twenty‐four patients (18 m, 6 f; age: 27 ± 8 y) were examined after a mean follow‐up of 34 ± 10 months. PTS was reduced from 15.2° ± 2.4° to 5.7° ± 3.8°. Scores at final follow‐up: IKDC 75.5 ± 1.5, Lysholm 79.9 ± 12.7, KOOS 77.5 ± 11.5, TAS was 5 (interquartile range 4–7). Postoperative Pain was significantly reduced (VAS: 4.0 ± 2.8 vs. 1.4 ± 1.3; *p* < 0.001). Compared to the contralateral side, ATT was higher in the operated knee (2.5 ± 2.9 mm; *p* = <0.01). Four patients underwent revision surgery (2x non‐traumatic instability; 2x traumatic ACL rupture). Additionally, 75% of patients returned to sports, while 64.3% of patients could return to their prior level.

**Conclusion:**

Combined slope‐reducing HTO and ACLR lead to good clinical outcomes, high patient satisfaction, and a low failure rate. Patients were able to return to activity, but often at a lower sports level. Anterior tibial translation remains slightly increased compared to the healthy side.

**Level of Evidence:**

Therapeutic study Level IV, case series.

**Abbreviations**
ACL: anterior cruciate ligament
ACLR: anterior cruciate ligament reconstruction
ATT: anterior tibial translation
HTO: high tibial osteotomy
IKDC: the International Knee Documentation Committee Score
IQR: interquartile range
KOOS: the Knee Osteoarthritis and Outcome Score
PCL: posterior cruciate ligament
PROMs: patient reported outcome measures
PTS: posterior tibial slope
SSD: side‐to‐side‐difference
TAS: Tegner Activity Scale
VAS: visual analogue scale

## INTRODUCTION

The annual incidence of anterior cruciate ligament (ACL) tears is approximately 46/100,000 [21]. After primary ACL reconstruction (ACLR), between 5% and 25% of patients experience reinjury or instability. In cases of revision surgery, the failure rate is even higher, ranging from 3.5% to 33%, compared to primary ACLR [4, 13]. Several factors contribute to ACL rerupture, with trauma being the most common cause for a first‐time rerupture of ACLR. However, in cases of multiple revisions, non‐traumatic, gradual‐onset injuries are more prevalent [8]. Important risk factors for rerupture include younger patient age and lack of neuromuscular control [3, 25].

Literature identified several other reasons for ACLR failure, which are primarily focused on biological factors and technical errors. These include malpositioning of bone tunnels and the use of allografts [37]. Risk factors for graft failure can be categorised into patient‐related, surgeon‐related and biological factors [33]. In recent years, there has also been increased focus on the bony alignment of the knee [37].

Especially the relationship between an elevated posterior tibial slope (PTS) and increased forces on the ACL was described as a significant risk factor. Biomechanical and clinical studies have demonstrated a linear association between a higher PTS and increased stress on the ACL [6, 18, 24]. Recent literature has determined that a PTS greater than 12° serves as a risk factor [28, 36]. Exemplary, Salmon et al. [28] showed an 11‐fold risk for ACL failure in adolescents with a PTS > 12° compared to an adult cohort with a PTS < 12° in their long‐term‐follow‐up.

Furthermore, Gwinner et al. [16] highlighted the dramatic impact of an increased PTS when considering multiple ACL failures. They observed that an increased PTS > 12° was associated with an odds ratio of 11.6 for multiple re‐ruptures in a mid‐term follow‐up cohort of 57 ± 61 months. Although defining an elevated PTS as one of the major risk factors for ACL retear, we lack evidence concerning the clinical outcome after slope reducing high tibial osteotomy (HTO) in combination with revision ACLR [2, 23]. Some previous studies revealed a trend for good patient satisfaction and clinical results for slope reducing HTO in combination with revision ACLR [2, 9, 30, 31], however the number of patients and the follow up period were limited.

The aim of the study was to evaluate the clinical results after slope reducing HTO in combination with revision ACLR. It was hypothesised that slope reducing HTO in combination with revision ACLR leads to good clinical results with low failure rate, high patient satisfaction, high return to sports rate but limited return to high impact sports.

## MATERIAL AND METHODS

The present bicentric outcome study of retrospectively collected data has been approved by an Institutional‐Review‐Board (2022‐527‐S‐NP). Patients aged older than 18 years who underwent slope reducing anterior closed wedge HTO combined with revision ACLR for ACL‐insufficiency with a posterior tibial slope > 12° between 11/2016 and 01/22 with a minimum follow‐up of 24 months after the revision ACLR were included. Exclusion criteria were rheumatoid arthritis, osteoarthritis > Kellgren & Lawrence Grade II, or medial open wedge high tibial osteotomy with additional slope reduction.

### Radiological measurement

The PTS was measured pre ‐and directly postoperatively on strict lateral x‐rays according to the method by Dejour et al. [10]. As described, two circles were placed 5 cm and 10 cm distal to the joint line into the tibial shaft. The proximal anatomical tibial axis was defined by the line crossing the centre of both circles. A horizontal line was placed perpendicular to the anatomical axis. A tangential line was then drawn in the middle of the lateral and the medial tibial plateau to represent the dorsal tibial inclination. The PTS was then defined as the angle between the horizontal and the tangential line. According the works of Lee et al. [22] and Webb et al. [34] the normal values of the PTS should not exceed the value of 12°. According to Dror Paley the PTS ranges between 6° and 13° [26].

### Surgical technique

**Figure 1:**
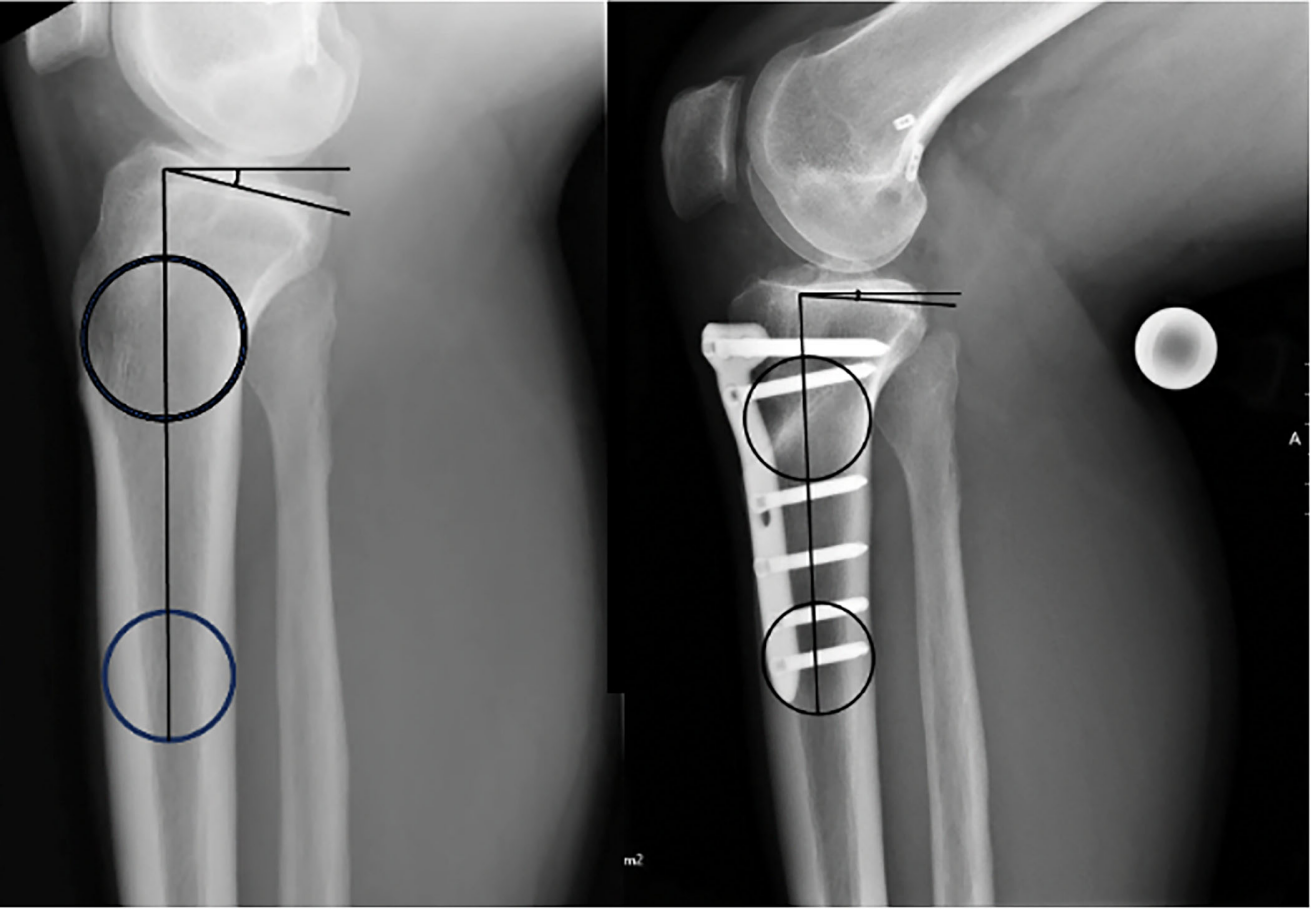
Preoperative (24.5° PTS) and postoperative (7.0° PTS) radiographs before and after slope reducing high tibial osteotomy. PTS, posterior tibial slope.

The slope reducing HTO was performed as previously described by Petersen et al. [17]. Slope reducing HTO was performed in patients with an elevated PTS > 12° when they suffered more than one failure of ACLR. Slope reducing HTO was also performed, when first time ACLR failure happened, and the patient's PTS was >15°. The patient was placed in supine position in general anaesthesia. Diagnostic arthroscopy was performed and afterwards an infratuberosity anterior closing wedge HTO was performed. An 8–10 cm longitudinal skin incision was made 1–2 cm medial to the tibial tuberosity. A k‐wire is placed just inferior to the tibial tuberosity aiming at the tibial insertion of the posterior cruciate ligament (PCL). A second k‐wire was placed distal to the first one also aiming at the insertion of the PCL. The distance between the two k‐wires at the anterior tibial cortex defined the height of the osteotomy wedge and was planned preoperatively according to the desired correction (approximately 7°). The tibia was then cut along the k‐wires, the bone wedge removed, and the osteotomy closed by gentle axial pressure. The bone was then stabilised with an angular stable plate (TomoFix, Depuy Synthes, J&J MedTech, New Brunswick, New Jersey) (Figure 1). In case of widened bone tunnels of the previous ACLR in anatomic or partial anatomic position, simultaneous filling of the tunnels was performed. Therefore, the tunnels were prepared until the sclerotic zone was fully removed and then filled with allogeneic spongious bone. After complete healing of the osteotomy the removal of the osteosynthesis material was performed alongside the ACLR after approximately one year.

All revision ACLR were performed in a single bundle, anatomic technique [1]. The femoral tunnel placement was performed via the anteromedial portal. The ipsilateral quadriceps was the preferred graft. A suspensory fixation system (Tight Rope, Arthrex, Naples, FL) was used for femoral fixation, and for the tibial fixation one centre used an interference screw and the other centre used a monocortical screw fixation. If required, meniscus surgery was performed using either all‐inside repair or inside‐out sutures according to the configuration of the meniscus tear. In one centre anterolateral ligament (ALL) reconstruction was performed, the other centre preferred a lateral extraarticular tenodesis. If a lateral extraarticular tenodesis was performed, it was performed after the Lemaire technique [20]. For the Lemaire technique a 10 mm stripe of the iliotibial band was cut over a length of approximately 8 cm. It was passed beneath the lateral collateral ligament (LCL) and fixed femoral with a bio‐interference screw posteriorly and proximally to the lateral epicondyle. If anterolateral ligament reconstruction was performed, the surgery was done as described in the following paragraph: The gracilis tendon was harvested and armed with non‐absorbable sutures. Stab‐incisions at the femoral and tibial insertion sites were performed. Transection of the iliotibial tract at the femoral site was performed. The femoral insertion was detected by palpating the dorsally and proximally descendent epicondyle. Two convergent 4 mm drill holes were performed at the tibial insertion site of the ALL. They were connected through an appropriate instrument and therefore a semicircular tunnel was created. At the femoral insertion point, a guide wire from lateral to medial was positioned and overdrilled with a double gracilis‐tendon‐sized drill, usually 5 mm. A nitinol wire was positioned for the later fixation. The gracilis‐graft was press‐fit shuttled through the tibial tunnel. The two graft ends were then passed under the iliotibial tract to the femoral incision and pulled into the femoral channel using the mentioned guide wire. Both graft ends were fixed in the femoral channel at 0° of extension and 0° of rotation using an interference screw in the same or +1 mm size of the femoral channel via the nitinol wire [32].

### Postoperative rehabilitation

After the HTO, two weeks of partial weight bearing were indicated. No brace was used. After revision ACLR the patient was put in a hinged brace for 6 weeks. Patients obtained a restricted range of motion in case of concomitant meniscus repair (6 weeks 60° for radial and root tears; 90° for any other). Partial weight bearing was also depending on a performed meniscus suturing; Patients with radial or root tears were not allowed to put weight on their operated leg whereas all others were advised two weeks of partial weight bearing with 20 kilograms.

Six weeks postoperatively, patients aimed to focus on muscular strengthening; eight weeks after surgery cycling was allowed and after 12 weeks patients were allowed to run. High risk sports with high pivoting forces, such as football or tennis were not allowed before nine months after surgery.

### Clinical evaluation

At a minimum follow‐up of 2 years postoperatively after the ACLR, patients were invited for a clinical examination. Range of motion, anterior and posterior translation of the tibia and pivot shift was documented. The Rolimeter device (Aircast Europa, Neubeuern, Germany) was used to assess side‐to‐side differences (SSD) of the anterior tibial translation (ATT) in millimetre. Additionally, a questionnaire was completed which included patient reported outcome measures (PROMs) as well as questions about the patients' satisfaction with the postoperative results (1 = very satisfied, 5 = very unsatisfied) and about their subjective knee stability. In terms of the PROMs, the pre ‐and postoperative pain according to a visual analogue scale (VAS pain) was evaluated; the International Knee Documentation Committee Score (IKDC score), the Lysholm score, and the Knee Osteoarthritis and Outcome Score (KOOS) were evaluated at the final follow‐up only. Additionally, revision surgeries were recorded.

### Return to sport

Patients were asked to complete a custom questionnaire about their sports habit before their first ACL injury and after slope‐reducing HTO with revision ACLR. Questions about the specific type of activity and about the time spent (hours per week) for sports activities were included. Additionally, the Tegner Activity Scale (TAS) was obtained.

### Statistical analysis

The present study was concepted as a retrospective cohort study, all patients undergoing combined slope reducing HTO and revision anterior cruciate ligament reconstruction for ACL‐insufficiency with a posterior tibial slope > 12° were included. Descriptive statistics were used to summarise categorical and continuous variables, with categorical variables reported as counts and percentages, and continuous variables reported as mean ± standard deviation or median and the interquartile range (IQR). Continuous variables were analysed using a Kolmogorov‐Smirnov Test to evaluate their distribution. Depending on the result, mean ± standard deviation or median and the IQR were used to demonstrate those variables. For group comparisons either a paired T‐Test or the Wilcoxon‐*U*‐test were utilised. Statistical significance was defined as *p* < 0.05. The statistical analysis was performed using the SPSS‐software version 26.0 (IBM‐SPSS, New York, USA).

## RESULTS

**Figure 2:**
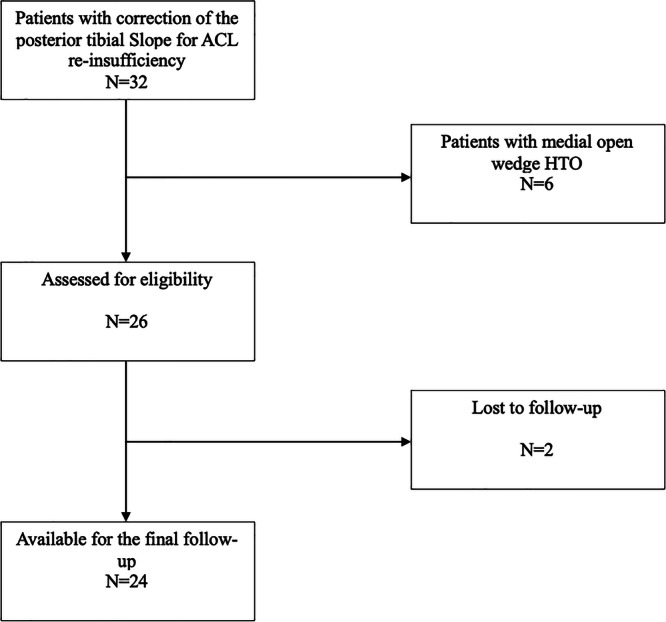
Flowchart of the eligible patients. ACL, anterior cruciate ligament; HTO, high tibial osteotomy.

A total of 24 (92.3%) of 26 eligible patients were available for inclusion in the final analysis. Two patients were not available and were lost to follow‐up Figure 2. Among the included patients, the mean PTS was reduced from 15.2 ± 2.4° to 5.7 ± 3.8° (*p* < 0.001). All patients received surgical treatment over two steps with revision ACLR one year after the initial surgery simultaneously with hardware removal. Eight patients received their second ACLR, 15 patients underwent their third ACLR and one patient underwent their fourth ACLR. Demographic and surgical characteristics of the patient population are provided in Table 1.

**Table 1:** Demographic and surgical characteristics of the 24 included patients.

Sex (*N*)		
Male	18	
Female	6	
Age (years)	27.4	±8.1
Body mass index (kg/m^2^)	25.9	±3.4
Follow‐up (months)	34.3	±10.4
Affected site (*N* (%))		
Right	11	(48.8%)
Left	13	(54.2%)
ACL graft insufficiency (*N* (%))		
Primary graft insufficiency	8	(33.3%)
Graft reinsufficiency	15	(62.5%)
Graft rereinsufficiency	1	(4.2%)
Graft type used for ACL revision (*N* (%))		
Quadriceps tendon ipsilateral	14	(58.3%)
4‐fold hamstring Tendon contralateral	3	(12.5%)
Allograft	3	(12.5%)
4‐fold hamstring Tendon ipsilateral	2	(8.3%)
Quadriceps tendon contralateral	2	(8.3%)
Concomitant surgeries (*N* (%))		
Anterolateral reconstruction/lateral extraarticular tenodesis	16	(66.7%)
Partial resection of the medial meniscus	1	(4.2%)
Medial meniscus repair	6	(25%)
Partial resection of the lateral meniscus	2	(8.4%)
Lateral meniscus repair	6	(25%)

Abbreviation: ACL, anterior cruciate ligament.

### Failures/complications

Four patients (16.7%) had undergone revision surgery at the time of the follow‐up (range 24–37 months). Of these four patients, two patients experienced an adequate trauma (foul during a football game, skiing accident) leading to a rerupture of the ACL graft, whereas two complained about atraumatic instability.

### Clinical outcome

Excluding the failures, 60% of patients were very satisfied with the surgical result, 25% were happy, 10% were neutral about their surgery and 5% were not satisfied with the surgical result. Pain was statistically significant reduced from 3.7 ± 2.5 preoperatively to 1.7 ± 1.7 postoperatively (*p* < 0.01) at a minimum follow‐up of 24 months (41.8 ± 11.8; range 25–69 months). The results of the PROMS are presented in Table 2.

**Table 2:** Clinical outcome in terms of PROMs at final follow up.

Clinical outcome in terms of PROMs	
Score	Outcome
IKDC score	75.5 ± 1.5
Lysholm score	79.9 ± 12.7
KOOS	77.5 ± 11.5
Symptoms	75.7 ± 13.9
Pain	87.2 ± 9.9
Activities of daily living	94.7 ± 6.7
Sports/recreation	75.2 ± 21.8
Quality of living	54.5 ± 16.1

Abbreviations: IKDC, International Knee Documentation Committee; KOOS, Knee Osteoarthritis and Outcome Score; PROMs, patient‐related outcome measures.

### Clinical examination

In the clinical examination, all patients had a full range of motion. Eleven patients (45.8%) showed increased extension postoperatively up to 15° of hyperextension (5°–15°). Regarding the Lachman‐test, in 68.8% of patients a firm endpoint was observed, in 25% of patients a soft endpoint was observed and in 6.2% of cases a prolonged anterior tibial translation was observed. In 12.5% of patients a Grade 1 pivot shift was noted and in 6.2% a Grade II pivot shift was eminent. There was a significant difference of ATT in the Rolimeter testing with a mean SSD of 2.5 ± 2.9 mm (*p* < 0.01).

### Return to activity

At time of follow‐up, 15 patients (75%) were sportively active, thereof 64.3% were able to return to their prior sports (Table 3). However, 37.5% of patients were able to return to high impact sports. No statistically significant difference was observed in the volume of sports practicing hours per week (preoperatively 2.25 ± 6.9 hours/week vs. 2.8 ± 7.1 hours/week; *p* = 0.325). The median TAS was 5 (IQR 4–7) at follow‐up.

**Table 3:** Sports involvement before the first ACL‐injury and at time of the follow‐up.

Sports involvement before the first ACL injury and at follow‐up^a^		
	Number of sportively active patients:	
	pre‐operation	post‐operation
General sports activity:	14	15
Low impact sports^b^:	6	12
High impact sports^c^:	8	3

Abbreviation: ACL, anterior cruciate ligament.

^a^ Minimum 24‐month‐follow‐up after combined slope‐reducing high tibial osteotomy and ACL revision;

^b^ Jogging, fitness, cycling;

^c^ Soccer, handball, basketball, skiing, combat sports;

## DISCUSSION

The major finding of present study was that patients undergoing slope‐reducing HTO and revision ACLR achieve favourable clinical results with high patient satisfaction and good knee stability. Additionally, return to sport rates can be achieved in a high number of patients, however the return to high impact sports was limited.

In our cohort, we preferred a two‐stage procedure to begin with slope reducing HTO and plate removal combined with revision ACLR after full consolidation of the osteotomy. We assumed that similar to the study of Mehl et al. [24], where isolated HTO to address varus osteoarthritis in ACL deficient knees could improve pain scores and knee stability, isolated slope decreasing HTO might decrease subjective knee stability. However, there exists no data yet confirming this assumption.

Several biomechanical studies emphasised the impact of the PTS on ACL graft forces. Slope reducing osteotomy may reduce the ACL graft forces of approximately 33% [18, 19]. Accordingly, the clinical impact of an increased PTS has been demonstrated as well.

When analysing cohorts of patients suffering from primary ACL injuries and comparing them to ACL intact patients, an increased PTS > 12° was observed more frequently [35, 36]. Especially in case of revision ACL, recent studies revealed a high impact of an increased PTS on multiple ACL failures [5, 16, 36]. Beel et al. [5] were able to demonstrate an increased PTS in 35% of ACL revision cases. Furthermore, multiple failures of the ACL were associated with an elevated PTS and the absolute measured values of the PTS were also increased in comparison to single ACL failure.

As it is proven that an increased PTS is a relevant risk factor, especially for multiple failures after ACLR, slope‐reducing HTOs have increasingly gained attention in recent years. Biomechanical studies observed significant reduction of force on the ACL graft after performing a slope‐reducing HTO [19].

Additionally, recent literature revealed beneficial clinical outcome and restoration of knee stability after slope‐reducing HTO and revision ACLR similar to our study [2, 31]. However, the number of patients was limited while studies have been performed on small cohorts [2, 9, 30, 31]. The largest cohort published by Akoto et al. [2] reported superior results compared to present study. The authors evaluated 20 patients after slope reducing HTO, revision ACLR and additional lateral extra‐articular tenodesis. Patients achieved 90.9 ± 6.4 points in the Lysholm Score, 95.2 ± 8.4 points in the KOOS Symptoms Section, 94.7 ± 5.2 points in the KOOS Pain section, 98.5 ± 3.2 points in the Activities of daily Living Section, 86.8 ± 12.4 points in the Sports/Recreation section and 65.4 ± 14.9 points in the Quality of Living section. Further studies demonstrated comparable results to the present study with the Lysholm score ranging from 73 to 89 and a IKDC score between 71 and 79 [9, 30, 31].

Additionally, the results of the present study demonstrated a low failure rate in terms of rerupture or re‐instability in a cohort with mainly multiple revision ACL surgery when referring to failure rates of up to 33% for revision ACLR in general [13]. In contrast, the failure rate is rather comparable to those after primary ACLR. Paterno et al. [27] described a failure rate of 29% of primary ACLR within a sportive, young cohort. Other literature revealed failure rates for primary ACLR between 3%–14% [11] and 5%–25% [13]. On the other hand, other authors were able to demonstrate more successful results with no required revision surgery for patients undergoing slope‐reducing HTO and revision ACLR [2, 9, 30, 31].

With regards to return to sports after slope‐reducing HTO, especially when analysing the ability to return to prior activity, evidence is scarce. Authors of recent studies observed postoperative TAS around 7 which is slightly superior to our results [2, 9, 31]. Though, when generally comparing the return to sports rates after revision ACLR or primary ACLR, results are similar to our study [4, 12, 29]. Sepulveda et al. [29] evaluated patients after primary ACLR and observed a postoperative return to sports rate of 81%. However, only 65% of patients were capable of returning to their preinjury sports and only 55% managed to return to a competitive level indicating a strong limitation of returning to high demanding sports even after first time injury. Similarly, we noted high return to sports but higher limitation in participating in high impact sports. In this study patients were obliged to adapt their level of sports participation.

To sum up, favourable patient outcome was achieved after slope‐reducing HTO. According to the existing literature, the indication for slope‐reducing HTO is based on ACL re‐insufficiency and a PTS > 12° [7, 16, 28, 34, 36]. However, this value of 12° is solely based on clinical studies evaluating the risk of ACL re‐insufficiency. Hence, further clinical studies evaluating the postoperative results are needed in order to understand which patients benefit most from slope reducing HTO. This may help to define clear indications for sagittal correction of the proximal tibia.

## LIMITATIONS

First, measuring the PTS was performed using the method according to Dejour et al. [10]. Current literature does not outline a standardised version for the correct evaluation of the PTS. According to the study of Faschingbauer et al. [14] the PTS might have been overestimated in some cases. Further, the expectation of strict lateral X‐rays of the tibia was not met in every case.

Third, the number of eligible patients in this study was limited.

Fourth, the included patients were examined after a short‐term follow‐up not focusing on mid ‐or long‐term‐rerupture rate as well as on the development of osteoarthritis. Further, when analysing the concomitant surgical procedures, patients underwent heterogenous procedures. In contrast to that, especially in revision surgery additional meniscus surgery is present in a high number of cases [15].

Lastly, due to the limited number of patients and the lack of preoperative values in terms of PROMs the study has to be considered the analysis of a trend. Further investigation based on prospectively designed studies has to be performed.

## CONCLUSION

Slope reducing HTO and revision ACLR for recurrent ACL insufficiency with an increased PTS > 12° trends to lead to acceptable outcome and favourable patient satisfaction. Patients were able to return to activity, but mainly at lower sports level after surgery. Anterior tibial translation remains significantly increased compared to the healthy side. However, further investigation needs to be done.

## AUTHOR CONTRIBUTIONS

All authors contributed to the study conception and design. Material preparation, data collection and analysis were performed by Lorenz Fritsch, Stefan Hinterwimmer MD, Julian Mehl MD and Konstantin Dworschak. The first draft of the manuscript was written by Lorenz Fritsch and all authors commented on previous versions of the manuscript. All authors read and approved the final manuscript.

## CONFLICT OF INTEREST STATEMENT

The authors declare no conflicts of interest.

## ETHICS STATEMENT

Approval by the ethics committee of the Technical University Munich was obtained (2022‐527‐S‐NP). The study complied with the Declaration of Helsinki and its respective amendments. Informed consent was obtained from all individual participants included in the study. Patients signed informed consent regarding publishing their data.

## Data Availability

The data sets used and/or analysed during the current study are available from the corresponding author on reasonable request.
